# Factors Influencing the Desirability, Acceptability, and Adherence of Patients with Diabetes to Telemedicine

**DOI:** 10.3390/medicina58080997

**Published:** 2022-07-26

**Authors:** Raul Patrascu, Alin Albai, Adina Braha, Laura Gaita, Sandra Lazar, Ovidiu Potre, Bogdan Timar

**Affiliations:** 1 Department of Functional Sciences, “Victor Babes” University of Medicine and Pharmacy, 300041 Timisoara, Romania; patrascu.raul@umft.ro; 2 “Pius Brinzeu” Emergency Hospital, 300723 Timisoara, Romania; braha.adina@umft.ro (A.B.); gaita.laura@umft.ro (L.G.); bogdan.timar@umft.ro (B.T.); 3 Second Department of Internal Medicine, “Victor Babes” University of Medicine and Pharmacy, 300041 Timisoara, Romania; sandra.lazar@umft.ro; 4 First Department of Internal Medicine, “Victor Babes” University of Medicine and Pharmacy, 300041 Timisoara, Romania; potre.ovidiu@umft.ro; 5 Centre for Molecular Research in Nephrology and Vascular Disease, “Victor Babes” University of Medicine and Pharmacy, 300041 Timisoara, Romania

**Keywords:** telemedicine, diabetes care, eHealth, healthcare policies

## Abstract

*Background and Objectives:* Telemedicine solutions have proven their value and efficacy in augmenting diabetes care. In addition to the availability of tools needed to implement telemedicine solutions for patients with diabetes, the patients’ desirability, acceptance, and adherence represent major burdens in implementing them. The main aim of this research is to evaluate which factors are influencing the desirability, acceptance, and adherence of patients with diabetes to telemedicine interventions in diabetes care. *Materials and Methods:* QTelemeDiab, a previously validated instrument for assessing patients’ desirability, acceptance, and adherence to telemedicine in diabetes care, was used on 114 enrolled patients with diabetes mellitus, in parallel with demographic, socio-economic, disease history, and psychometric data from all patients. *Results:* Left-skewed score distributions were observed for the QTelemeDiab total score (median = 166; skewness = −1.738), as well as all its components, thus denoting a high desirability, acceptance, and adherence towards telemedicine use. The presence of severe depression was associated with significant decreases in the QTelemeDiab score (148 vs. 167; *p* < 0.001), as well as on the desirability sub-score (101 vs. 115; *p* < 0.001) and adherence sub-score (30 vs. 35; *p* < 0.001). The presence of severe anxiety was associated with significant decreases in QTelemeDiab score (150 vs. 166), as well as the desirability sub-score (104 vs. 114; *p* = 0.008) and adherence sub-score (30 vs. 34; *p* = 0.012). *Conclusions:* There is a high desirability, acceptance, and adherence to the use of telemedicine interventions in patients with diabetes, both in special and in normal epidemiological settings. The presence of severe anxiety decreases the patient’s desirability, acceptance, and adherence, while the presence of severe depression decreases the patient’s desirability and adherence to the use of telemedicine interventions in diabetes care.

## 1. Introduction

Diabetes mellitus is presently a major public health issue as an estimated 537 million people currently live with diabetes worldwide and the total diabetes-related health expenditure is estimated to reach one trillion USD in 2030. Furthermore, a continuous increase in diabetes prevalence and associated diabetes-related health expenditure is estimated in the coming years [1]. Delivering optimal care for these patients leads to significant improvements in patients’ prognoses, as well as to major decreases in indirect diabetes-related costs for society. Considering the high prevalence of diabetes and its increasing trend, as well as the limited healthcare resources—particularly the limited human resource—delivering quality care for people with diabetes is, and will be, a challenge for healthcare systems worldwide [2].

The World Health Organization defines telemedicine as being “*The delivery of health care services, where distance is a critical factor, by all health care professionals using information and communication technologies for the exchange of valid information for diagnosis, treatment and prevention of disease and injuries, research and evaluation, and for the continuing education of health care providers, all in the interests of advancing the health of individuals and their communities*” [3]. Telemedicine thus rises as a possible solution to optimize the flow of care in patients with diabetes [4].

Considering some of the particularities of diabetes care, with the disease being a chronic condition, which leads to a long-term relationship between patients and healthcare professionals, its care can be augmented through the use of several technological devices. Many such devices are becoming part of regular care delivery (i.e., sensor-augmented insulin pumps, smart glucose meters, smart insulin-delivering pens, electronic food diaries) in diabetic patients [4]. Thus, diabetes care has become a scenario in which telemedicine rises as a valuable intervention, leading to improvements in both care delivery and patient outcomes, while optimizing the healthcare flow and reducing diabetes-related costs [5]. Several interventional scenarios are possible for telemedicine usage in diabetes: in chronic patients with stable disease, telemedicine may be used to replace the in-person visit to the outpatient clinic; telemedicine may be used for remote glycemic control, diet pattern, and insulin dosage monitoring; as well as for delivering medical advice during acute events (i.e., hypo- or hyper-glycemia) [6,7,8].

Regarding the use of telemedicine in diabetes, there are two main barriers: access to technology, and the patients’ desire, acceptability, and adherence to the usage of telemedicine tools for their respective care [9,10,11,12,13]. However, the beneficial effects of using telemedicine as an addition to classical medical visits are already proven and have been brought, once again, to specialists’ attention, with the onset of the COVID-19 pandemic. This favorable impact is not only related to improvements in glycemic control, and other cardiometabolic risk factors, but also to the importance of a constant and reassuring patient–healthcare-professional communication, especially since diabetes is known to be influenced by, and to influence, mental health [14,15,16]. Moreover, regarding mental health, the COVID-19 pandemic was proven to be associated with an increased incidence of anxiety and depression, with these conditions being even more pronounced in patients with chronic illness [17,18,19,20,21,22].

In this context, and with the development in early April 2020 of a new telehealth platform, *telediabet.ro*, freely available for every patient from Romania diagnosed with diabetes mellitus, an opportunity of offering a complex, yet simple technological solution for these individuals has emerged, from the necessity of identifying and analyzing factors, including those related to mental health, such as anxiety or depression, which could improve their experience. Therefore, the main aim of this study was to evaluate the patients’ desirability, acceptability, and adherence to telemedicine interventions in diabetes care and the parameters that could influence these characteristics.

## 2. Materials and Methods

### 2.1. Study Design

In this non-interventional, cross-sectional, consecutive-case, population-based study, 114 patients with both type 1 and type 2 diabetes were enrolled. According to the sample size estimation, to achieve a confidence level of 95% in parallel with a statistical power of at least 80%, 110 patients were needed. The study design, data collection and manipulation, and patients’ informed consent, were approved by the Ethics Committee from the “Pius Brinzeu” Emergency County Hospital, in Timisoara. The study was conducted according to the Helsinki statement of ethical principles for medical research.

### 2.2. Patient Recruitment

The enrolled patients were recruited using the *telediabet.ro* telemedicine platform, as well as from the Clinical Center for Diabetes, Nutrition and Metabolic Diseases, in “Pius Brinzeu” Emergency County Hospital, Timisoara, Romania. All patients had previously agreed to receive further invitations to participate in research. Patients’ participation in the study was voluntary and anonymous, with no collection of any participant-identification data. Therefore, the decision to participate in the study did not affect the access to, or quality of, the medical care provided. The consent form was included in the electronic form and was a sine qua non condition for enrollment in the study.

### 2.3. Patient Assessment

The desirability, acceptability, and adherence to telemedicine solutions of patients with diabetes was assessed using the QTelemeDiab instrument. QTelemeDiab is a previously validated instrument designed to evaluate the three previously mentioned items regarding telemedicine use and was designed specifically for patients with diabetes [11]. The QTelemeDiab questionnaire has 9 sections, with multiple items, all scored on a Likert scale (from the lowest value: 1 (not likely at all) to the highest: 5 (very likely)). The three dimensions were evaluated as follows:

- The desirability sub-score was the sum of Q6–Q8 with a score ranging from 24 to 120;
- The acceptability sub-score was the sum of Q1–Q5 with a score ranging from 5 to 25;
- The adherence sub-score was the sum of Q3, Q9 (minimum score: 8, maximum score: 40);
- The total scores ranged from 34 to 185.

A higher score is associated with a higher desirability, acceptability, and, consequently, adherence to telemedicine use in diabetes care.

Additionally, the QTelemeDiab instrument includes queries related to patients’ socio-demographic characteristics and medical history (including specific diabetes-related history), used in analysis to identify patterns in patients more prone to use telemedicine in diabetes care.

Data regarding the presence and severity of depression and anxiety were assessed using the PHQ-9 and GAD-7 instruments, respectively. The PHQ-9 questionnaire is a shortened version of the Patient Health Questionnaire, used to quantify depression severity [23]. The GAD-7 questionnaire was applied to studied patients in order to assess the presence and degree of anxiety [24]. Translated versions of both PHQ-9 and GAD-7 questionnaires were previously validated in the Romanian population.

The PHQ-9 scores of 4, 9, 14, 19, and 27 represent cut-points for: absent or minimal, mild depression, moderate depression, moderately severe depression, and severe depression, respectively [23]. GAD-7 scores of 5, 10, and 15 represent cut-points for mild, moderate, and severe anxiety, respectively [24].

### 2.4. Studied Sample Baseline Characteristics

The study sample included 114 patients: 30 men (26.3%) and 84 women (73.7%). The prevalence of diabetes among the group was 44.7% for type 1 and 37.7% for type 2. Most of the patients were employed, lived in rural areas, received an average income in 27.2% of cases, and had internet access multiple times per day, predominantly by using a smartphone. A total of 51 (44.7%) were diagnosed with diabetes more than 10 years ago. The general and anthropometric characteristics of the studied patients are presented in Table 1.

### 2.5. Statistical Analysis

Data were collected and analyzed using the Statistical Package for Social Sciences (SPSS) v.27 (IBM Corporation, Armonk, NY, USA). The results are presented as: average ± standard deviation (continuous variables with Gaussian distribution); median (interquartile range) (numerical variables with non-parametric distribution); and absolute and relative frequencies for categorical variables. The variables’ distributions were tested using the Kolmogorov–Smirnoff test (a *p*-value ≥ 0.05 described Gaussian distributions). The association between continuous variables was analyzed using the Spearman’s rho correlation coefficient, a higher-modulo rho being associated with a stronger association between variables.

The statistical significance of the associations was assessed using the unpaired Student’s-*t* test (continuous variables with Gaussian distribution) and Mann–Whitney U test (numerical variables with non-parametric distribution).

The sample size for this study was calculated during the study’s design, with prior enrolment of the patients calculated to obtain a statistical power of 80%, in parallel with a probability of: 1 − *alpha* = 0.05.

In this study, a *p* value lower than 0.05 was considered the threshold for statistical significance.

## 3. Results

In the study cohort, 64 (56.2%) of the patients were treated with insulin (35.1% with multiple daily injections, and 21.1% with insulin pumps). Most patients, 66 (57.9%), measured their glycemia multiple times per day, and only 23 (20.2%) checked their glycemia once daily. The continuous glucose monitoring systems were the devices most frequently used to evaluate glycemic control (39 individuals—34.2%), followed by the glucometers (accompanied by writing down the result in a diabetes journal) by 38 (33.3%). In the medical history, 47 (41.2%) had hospital admissions for diabetes-related emergencies and 27 (23.7%) had admissions due to hypoglycemia. Only 45 (39.5%) patients performed the HbA1c every 3 months, as recommended by the current guidelines. In terms of lifestyle, 50 (43.9%) patients reported exercising almost daily, while only 41 (36.0%) patients complied with the diet 5–7 days a week. In the studied group, 25 patients (21.9%) had signs of severe depression and 18 patients (15.8%) had signs of severe anxiety. The detailed characteristics of diabetes-related management in the studied sample are presented in Table 2.

**Figure 1:**
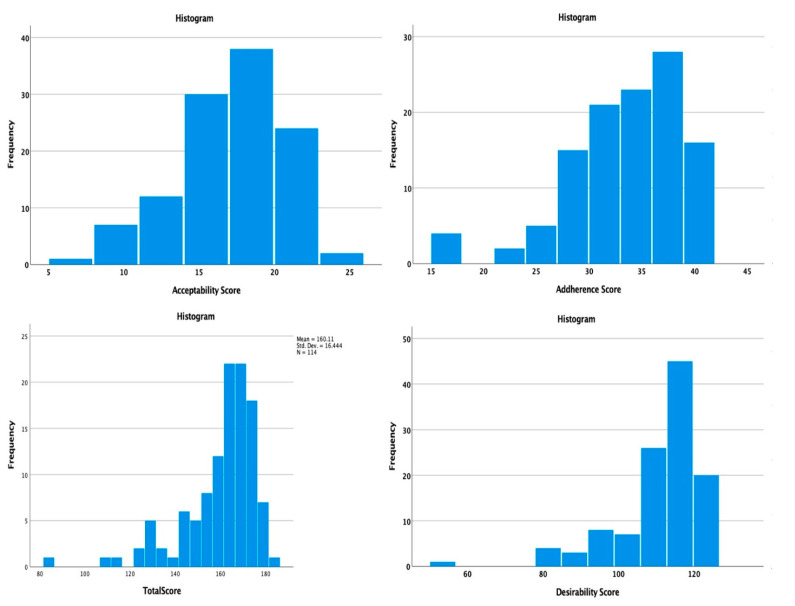
The distribution of desirability, acceptability, and adherence and total scores across studied group.

Left-skewed distributions (Figure 1) were observed for the total QTelemeDiab score (skewness = −1.738), as well as for all the sub-components (desirability = −2.048; acceptability = −0.558; adherence = −1.106) thus denoting a high desirability, acceptance, and adherence towards telemedicine use in patients with diabetes. The median, skewness, and kurtosis for QTelemeDiab scores and its sub-components is presented in Table 3.

**Figure 2:**
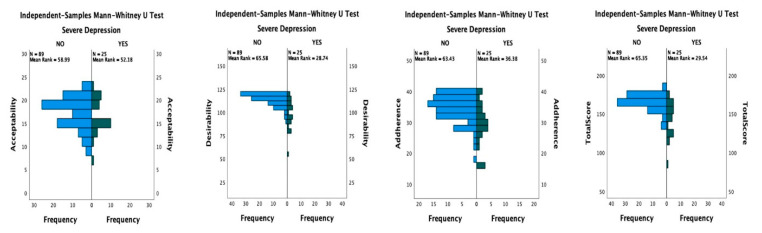
Distribution of desirability, acceptability, adherence, and total score, depending on severe depression.

The presence of severe depression was associated with significant decreases in the QTelemeDiab score (148 vs. 167; *p* < 0.001; Mann–Whitney U test), as well as on the desirability sub-score (101 vs. 115; *p* < 0.001; Mann–Whitney U test) and adherence sub-score (30 vs. 35; *p* < 0.001; Mann–Whitney U test), but had no significant impact on patients’ acceptability of telemedicine interventions in diabetes (18 vs. 15; *p* = 0.359; Mann–Whitney U test). The comparison of the QTelemeDiab score and its components, in patients with severe depression vs. patients without severe depression, is presented in Table 4 and the comparison of the score’s distribution is presented in Figure 2.

**Figure 3:**
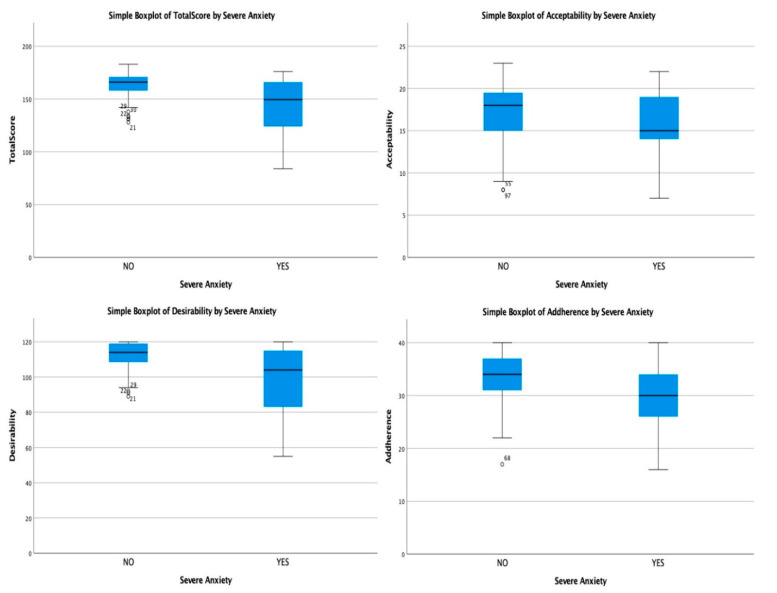
Boxplot diagrams: comparison of desirability, acceptability, adherence and total score, depending on severe anxiety.

The presence of severe anxiety was associated with significant decreases in QTelemeDiab score (150 vs. 166; *p* = 0.004; Mann–Whitney U test), as well as the desirability sub-score (104 vs. 114; *p* = 0.008; Mann–Whitney U test) and adherence sub-score (30 vs. 34; *p* = 0.012; Mann–Whitney U test). The presence of severe anxiety had no significant impact on the acceptability sub-score (15 vs. 18; *p* = 0.141; Mann–Whitney U test). The median comparison of QTelemeDiab scores with its sub-scores is presented in Table 5 and Figure 3.

When analyzing the statistical hypothesis that the distributions of the QTelemeDiab scores and its sub-scores (desirability, acceptability, and adherence) are influenced by several studied factors, using the non-parametric, independent-sample comparisons, it was observed that the QTelemeDiab score was significantly influenced by the presence of severe depression (148 vs. 167; *p* < 0.001; Mann–Whitney U test) and severe anxiety (105 vs. 166; *p* = 0.004; Mann–Whitney U test). The desirability score was significantly influenced by the presence of severe depression (101 vs. 115; *p* < 0.001; Mann–Whitney U test) and severe anxiety (104 vs. 114; *p* = 0.008; Mann–Whitney U test). The acceptability score was higher in the urban population than the rural population (18 vs. 15; *p* = 0.022; Mann–Whitney U test) and higher in patients who frequently monitored their HbA1c (17.5 vs. 13.5; *p* = 0.042; Kruskal–Wallis test). The adherence score was significantly lower in patients with severe depression (30 vs. 35; *p* < 0.001; Mann–Whitney U test) and in patients with severe anxiety (30 vs. 34; *p* = 0.012; Mann–Whitney U test). The statistical hypothesis analyses are presented in Table 6.

**Figure 4:**
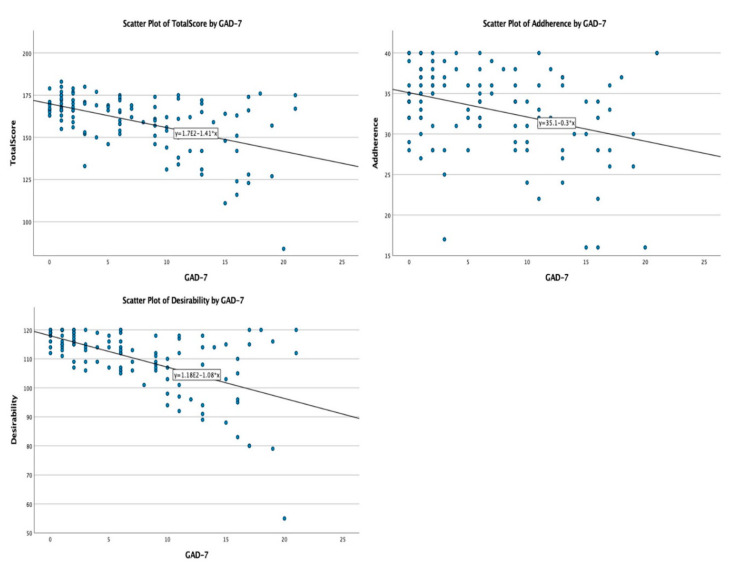
Correlations between the GAD-7 score and QTelemeDiab, adherence and desirability scores.

**Figure 5:**
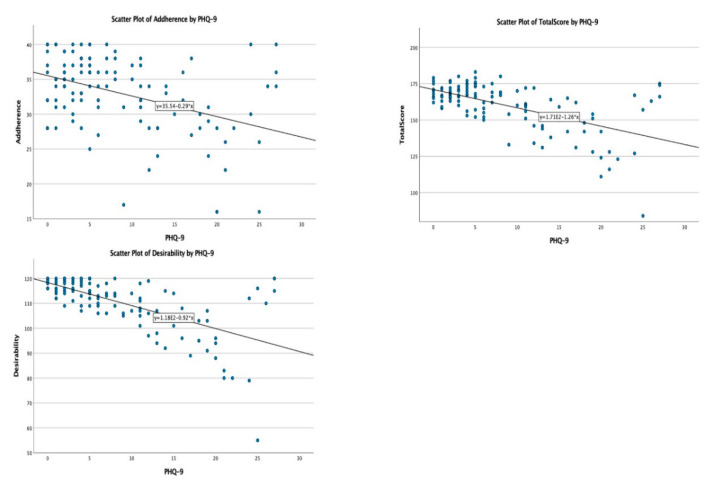
Correlations between PHQ-9 score and QTelemeDiab, adherence and desirability scores.

In the non-parametric correlation analysis conducted between the QTelemeDiab score, its sub-components, and factors having continuous, non-parametric variables’ distribution (Age, HbA1c value, PHQ-9 and GAD-7 scores), the following associations were observed: the GAD-7 score (Figure 4) was negatively, and significantly, correlated with desirability (Spearman’s rho = −0.569; *p* < 0.001), adherence (Spearman’s rho = −0.288; *p* < 0.001), and the QTelemeDiab score (Spearman’s rho = −0.447; *p* < 0.001); and the PHQ-9 score (Figure 5) was negatively, and significantly, correlated with desirability (Spearman’s rho = −0.623; *p* < 0.001), adherence (Spearman’s rho = −0.358; *p* < 0.001) and the QTelemeDiab score (Spearman’s rho = −0.524; *p* < 0.001).

## 4. Discussion

This research is one the first studies that aimed to evaluate the desirability, acceptability, and adherence of patients to the use of telemedicine solutions for the care of diabetes under special epidemiological conditions—the global COVID-19 pandemic—and the first study to assess the usage of telemedicine in Romanian patients with diabetes mellitus. It is known that patients with diabetes, as well as with other conditions frequently associated with diabetes, such as obesity or heart disease, are at a higher risk of developing moderate, severe, or even fatal forms of COVID-19 [12]. Therefore, the importance of telemedicine use is emphasized in these patients. *Telediabet.ro* is the first telemedicine platform implemented specifically for patients with diabetes, by the Diabetes Outpatient Clinic of the “Pius Brinzeu” Emergency County Hospital, in Timisoara, Romania. The telemedicine platform was developed and implemented from the first days of the special social and physical distancing measures enforced by the Romanian government. Using the telemedicine platform, any patient with diabetes was able to schedule a free-of-charge telemedicine consultation with a diabetes specialist, using augmented videoconferencing solutions, using a dedicated call-center, or using a pre-designed web-based questionnaire. During the nation-wide lockdown, the telemedicine platform had a peak usage of more than 200 weekly telemedicine consultations.

Considering the importance of telemedicine usage in the care of diabetes, both in special epidemiological conditions and during routine consultations—since telemedicine may enhance classical consultations and may optimize the delivery of care in these patients—and considering that the patients’ desirability, acceptance, and adherence to these solutions are the main barriers to the implementation of routine telemedicine care for patients with diabetes, there was a need for a study to evaluate these items in patients with diabetes [9,10,11]. Furthermore, the COVID-19 pandemic has been proven to strongly influence mental health, especially in patients with diabetes mellitus, since increasing knowledge—about the association of a more severe evolution of COVID-19, with high glycaemic values, and about DM being a very frequent comorbidity of COVID-19, with its complications being other risk factors—has brought fear and anxiety to these patients, in a context of unpredictability and uncertainty [17,18,19,20,21,22]. This has led to the idea of also assessing the impact of additional factors that could influence the usage of telemedicine, including psychological conditions.

Therefore, the present study used, as a main evaluation tool, the first validated instrument designed specifically to assess the desirability, acceptability, and adherence of patients with diabetes to telemedicine use in their medical care: QTelemeDiab. This instrument was previously validated for the population of patients with diabetes, both type 1 as well as type 2 [11].

Since this study was carried out during the COVID-19 pandemic, the patients’ responses may be skewed to higher desirability, adherence, and overall acceptability scores; therefore, a re-test will be performed during normal epidemiological conditions. In order to minimize this bias, as well as to gauge the usage of the QTelemeDiab instrument both under special as well as under normal epidemiological conditions, it was engineered to assess these components, with questions designed specifically for both situations. At the same time, the skewness of the responses’ distribution might have been influenced by the enrollment procedure, as a consequence of most of the enrolled patients being previous users of the telediabet.ro telemedicine platform. However, it should be stressed that this skewness of the distribution does not affect the study’s main objective, since factors associated with patients’ desirability, acceptance, and adherence to telemedicine usage can be properly identified and are themselves not influenced by the skewed score distribution. These factors are, therefore, being correctly assessed by the study [13].

## 5. Conclusions

There is a high desirability, acceptance, and adherence to the use of telemedicine interventions in patients with diabetes, both under special as well as under normal epidemiological conditions. In addition to the particularities of diabetes care, these characteristics are emphasizing the role and impact of telemedicine in the management of diabetes. The presence of severe anxiety decreases patients’ desirability, acceptance, and adherence, while the presence of severe depression only decreases patients’ desirability and adherence to the use of telemedicine interventions in diabetes care.

## Figures and Tables

**Table 1:** General characteristics of studied patients.

Studied Variable	N	Value (%)
**Gender**		
**Men**	30	26.30%
**Women**	84	73.70%
**Residence**		
**Urban**	26	22.80%
**Rural**	88	77.20%
**Occupation**		
**Employee**	70	61.40%
**Freelancer**	8	7.00%
**Retired**	29	25.40%
**Student**	4	3.50%
**Unemployed**	3	2.60%
**Education**		
**Primary School**	3	2.60%
**Highschool**	33	28.90%
**University degree**	40	35.10%
**Master or PhD**	38	33.30%
**Family Income**		
**Very low**	1	0.90%
**Low**	24	21.10%
**Average**	31	27.20%
**High**	26	22.80%
**Very high**	8	7.00%
**No answer**	16	14.00%
**Internet access**		
**Multiple times per day**	110	96.50%
**Sporadic**	4	3.50%
**Most used device for internet access**		
**PC/laptop**	29	25.40%
**Tablet**	3	2.60%
**Smartphone**	82	71.90%
**Diabetes category**		
**Type 1 diabetes**	51	44.70%
**Type 2 diabetes**	43	37.70%
**Patient of a child with type 1 diabetes**	20	17.50%
**Diabetes duration**		
**Less than 1 year**	5	4.40%
**1 to 5 years**	39	34.20%
**6 to 10 years**	19	16.70%
**More than 10 years**	51	44.70%
**Depression**		
**Without severe depression**	89	78.10%
**With severe depression**	25	21.90%
**Anxiety**		
**Without severe anxiety**	96	84.20%
**With severe anxiety**	18	15.80%

**Table 2:** Characteristics of patients’ diabetes management.

Studied Variable	N	Value (%)
**Diabetes treatment**		
**Oral antidiabetic drugs**	34	29.80%
**Oral and injectable non-insulin antidiabetic drugs**	4	3.50%
**Oral antidiabetic drugs and insulin**	8	7.00%
**Only diet**	4	3.50%
**Only insulin**	40	35.10%
**Insulin pump**	24	21.10%
**Glycemic monitoring**		
**Multiple times per day**	66	57.90%
**Once per day**	23	20.20%
**Once per week**	17	14.90%
**Once every few weeks**	5	4.40%
**Once every few months**	1	0.90%
**Never**	2	1.80%
**Device used for glycemic monitoring**		
**A glucometer accompanied by writing down the result in a diabetes journal/notebook**	38	33.30%
**A glucometer without writing down the result**	34	29.80%
**Continuous glucose monitors**	39	34.20%
**Laboratory tests**	1	0.90%
**No monitoring**	2	1.80%
**Admissions for diabetes**		
**Due to a very increased glycemic level**	27	23.70%
**Due to a hypoglycemia**	5	4.40%
**Due to a very increased glycemic level, due to a hypoglycemia**	3	2.60%
**Due to a diabetes complication**	11	9.60%
**Due to a diabetes complication, due to a hypoglycemia**	1	0.90%
**No admissions for diabetes**	67	58.80%
**HbA1c periodicity**		
**Once every 3 months**	45	39.50%
**Once every 6 months**	30	26.30%
**Once a year**	29	25.40%
**Once every few years**	9	7.90%
**Never**	1	0.90%
**Exercise**		
**5–7 days**	50	43.90%
**3–4 days**	22	19.30%
**1–2 days**	28	24.60%
**No exercise routine**	14	12.30%
**Diet**		
**1–2 days**	21	18.40%
**3–4 days**	43	37.70%
**5–7 days**	41	36.00%
**No healthy diet**	9	7.90%

**Table 3:** The characteristics of desirability, acceptability, adherence and total score to telemedicine in the studied group.

Component	Median [Interquartile Distance]	Skewness	Kurtosis
Desirability	114 [11]	−2.048	5.563
Acceptability	17 [4]	−0.558	−0.179
Adherence	34 [7]	−1.106	1.436
QTelemeDiab score	166 [17]	−1.738	3.967

**Table 4:** The comparison of desirability, acceptability, and adherence to telemedicine (plus total scores), depending on the presence of severe depression.

	Without Severe Depression (n = 89)	With Severe Depression (n = 25)	*p*
Acceptability	18 [15; 19]	15 [15; 19]	0.359
Desirability	115 [111; 119]	101 [89; 110]	**<0.001**
Adherence	35 [32; 37]	30 [26; 34]	**<0.001**
QtelemeDiab score	167 [160; 172]	148 [128; 162]	**<0.001**

The results are presented as continuous variables with non-parametric distributions. Results are presented as medians and [interquartile range]. *p*-values are calculated with the Mann–Whitney U test. Significant differences are marked in bold text (statistical significance threshold: *p* lower than 0.05).

**Table 5:** The comparison of desirability, acceptability, and adherence to telemedicine (plus total scores), depending on the presence of severe anxiety.

	Without Severe Anxiety (n = 96)	With Severe Anxiety (n = 18)	*p*
Acceptability	18 [15; 20]	15 [14; 19]	0.141
Desirability	114 [109; 119]	104 [83; 115]	**0.008**
Adherence	34 [31; 37]	30 [26; 34]	**0.012**
QTelemeDiab score	166 [158; 171]	150 [124; 166]	**0.004**

The results are presented as continuous variables with non-parametric distributions. Results are presented as medians and [interquartile range]. *p*-values are calculated with Mann–Whitney U test. Significant differences are marked in bold text (statistical significance threshold: *p* lower than 0.05).

**Table 6:** The null hypothesis test summary of the distribution of desirability, acceptability, adherence to telemedicine (plus total scores), of diabetic patients across influencing factors.

Factors Analyzed	*p*-Value for Comparison of Variable’s Distribution According to the Analyzed Factor			
	Desirability	Acceptability	Adherence	QTelemeDiab Score
Gender *	0.552	0.341	0.776	0.834
Residence *	0.626	**0.022**	0.107	0.149
Occupation **	0.908	0.612	0.193	0.879
Education **	0.056	0.162	0.647	0.498
Family income **	0.487	0.485	0.677	0.567
Diabetes category **	0.566	0.219	0.116	0.149
Glycemic monitoring **	0.553	0.472	0.657	0.589
Admissions for diabetes **	0.676	0.184	0.814	0.827
HbA1c periodicity **	0.168	**0.042**	0.200	0.081
Exercise **	0.763	0.798	0.887	0.846
Diet **	0.644	0.107	0.813	0.649
Severe Depression *	**<0.001**	0.359	**<0.001**	**<0.001**
Severe Anxiety	**0.008**	0.141	**0.012**	**0.004**

Significant differences in variables’ distributions are marked in bold text. * Independent-samples Mann–Whitney U Test; ** Independent-samples Kruskal–Wallis Test.

## Data Availability

Data available on request due to privacy restrictions. The data presented in this study are available on request from the corresponding author after the request’s approval by the hospital’s ethics committee. The data are not publicly available due to privacy restrictions, according to the hospital’s internal regulations.
